# Histological and molecular aspects of oral squamous cell carcinoma (Review)

**DOI:** 10.3892/ol.2014.2103

**Published:** 2014-04-29

**Authors:** CÉSAR RIVERA, BERNARDO VENEGAS

**Affiliations:** 1 Unit of Histology and Embryology, Department of Basic Biomedical Sciences, Faculty of Health Sciences, University of Talca, Talca 3460000, Chile; 2 Biomedical Sciences Master Program, Oral Pathology Mention, Faculty of Health Sciences, University of Talca, Talca 3460000, Chile; 3 Unit of Oral Pathology, Department of Dentistry, Faculty of Health Sciences, University of Talca, Talca 3460000, Chile

**Keywords:** mouth neoplasms, oral squamous cell carcinoma, oral cancer, p53, Ki-67, collagen type IV

## Abstract

Oral squamous cell carcinoma (OSCC) represents 95% of all forms of head and neck cancer, and over the last decade its incidence has increased by 50%. Oral carcinogenesis is a multistage process, which simultaneously involves precancerous lesions, invasion and metastasis. Degradation of the cell cycle and the proliferation of malignant cells results in the loss of control mechanisms that ensure the normal function of tissues. The aim of the current review is to present the histopathological features of OSCC, including potentially malignant changes, the international classification of tumors, the tumor invasion front and tumor biomarkers (Ki-67, p53, homeobox genes and collagen type IV), as well as the tumor microenvironment and function of cancer-associated fibroblasts in the most common type of oral cancer that is encountered by dental surgeons. In OSCC, associations have been identified between the proliferation, basal lamina degradation and connective tissue modulation. Therefore, the comparison of these factors with the survival time of OSCC patients from the histopathological diagnosis is of interest.

## 1. Introduction

Head and neck cancer is one of the 10 most common types of cancer worldwide, afflicting >500,000 individuals each year. Oral cancer is considered to be a preventable condition, due to the possibility of early detection and treatment (1). Oral squamous cell carcinoma (OSCC) represents 95% of all forms of head and neck cancer, and during the past decade its incidence has increased by 50% (2,3). Snuff and alcohol consumption are associated with 90% of patients that exhibit oral cancer (1) and the two factors appear to have a synergistic effect (4).

The majority of OSCC are diagnosed at a late phase (5), in stages III or IV (6,7), which markedly decreases the chances of survival and leads to a significant deterioration in patient quality of life.

Despite the currently available therapeutic strategies, which include the excision of malignant tissue and combination of radiotherapy and chemotherapy, the five-year survival rate is only 53% (3). In addition, a high percentage of patients have a poor response to therapy and high recurrence rates (8).

The purpose of the current review was to present the histological and molecular characteristics of the most common type of oral cancer encountered by dental surgeons.

## 2. Histology

In general, cancers, including OSCC, emerge from the accumulation of genetic changes and epigenetic anomalies in the signaling pathways that are associated with cancer, resulting in phenotypes that facilitate OSCC development. This process was summarized by Hanahan and Weinberg in ‘Hallmarks of Cancer’ (9).

**Figure 1:**
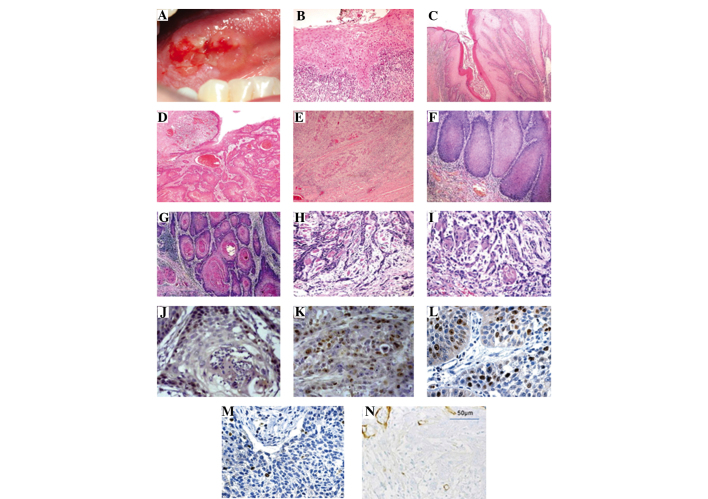
(A) Oral squamous cell carcinoma (OSCC) of the lateral edge of the tongue (13). (B) Severe dysplasia of the surface epithelium associated with chronic inflammatory infiltration at the stromal-epithelial interface of the dysplastic epithelium (stain, H&E; magnification, ×50) (13). Histological grades of tumor differentiation of OSCC: (C) Well-differentiated, hyperkeratosis and inflammation associated with the stromal-epithelial interface; (D) moderately differentiated; and (E) undifferentiated infiltrating and dispersed cells with no clear demarcation between the front and surrounding tissue invasion (stain, H&E; magnification, ×25) (13). Different patterns of invasion at the tumor invasion front according to the cell morphology: (F) Wide fronts of invasion (score 1); (G) islet cell widths (score 1); (H) thin infiltrating cords (score 2); and (I) individual cells invading the interface (score 3) (1). OSCC patients (J) with recurrence and (K) without recurrence. Antibody staining for Ki-67 with a high degree of nuclear staining (magnification, ×400) (16). Representative samples of homeobox protein, HOXB7 immunohistochemical expression in OSCC with (L) high and (M) low expression (32). (N) Immunohistochemical expression of type IV collagen α2 chain in undifferentiated OSCC (38).

OSCC is a malignant neoplasm derived from the stratified squamous epithelium of the oral mucosa (10). Its pathogenesis is multifactorial, associated with cigarette smoke, alcohol (11) and snuff, as well as the papilloma virus, among others (12). The malignant neoplasm occurs at various sites, the most frequent being the lip, lateral edges of the tongue (Fig. 1A) (13) and floor of the oral cavity. The incidence of OSCC increases with age, with the majority of OSCC occuring in patients >40 years (14).

OSCC is characterized by histopathological and clinical manifestations. All carcinogenesis evolves from initial cell injury to the formation of a malignant neoplasm (9). Histologically, the lesion passes through various phases (preneoplastic damage) until the ultimate formation of a cancer. This carcinogenesis may be associated with precancerous lesions (such as leukoplakia, erythroplakia and mixed). However, it is necessary to consider that not all reactional lestions or potentially malignant lesions result in the subsequent development of malignant neoplasms (15).

### Potentially malignant changes

According to their histological appearance, lesions that present in the epithelium during the process of carcinogenesis may be classified according to their reactive epithelial changes (such as hyperkeratosis, hyperplasia and acanthosis) or preneoplastic changes (including mild, moderate and severe dysplasia; Fig. 1B) (16) prior to the establishment of an invasive carcinoma (12,14,17). Oral cancer originates as an epithelial dysplasia and is characterized by the altered proliferation of dysplastic squamous cells on the surface of the epithelial layer, which subsequently degrades the subepithelial basement membrane (BM). Degradation of the BM results in local destruction and distant invasion via metastasis. Local invasion to the underlying tissue occurs via the islets and cords of epithelial cells (18).

The ability to metastasize is directly associated with the differential grade of tumor cells, similar to that of the neoplastic tissue architecture and normal epithelium (14).

### International Classification of Tumors (World Health Organization) and the tumor invasion front (TIF)

Currently, two systems are used to histologically classify tumor lesions; the International Histological Classification of Tumors (Fig. 1C–E) and the pattern of the TIF (19). The initial classification of lesions is based on the degree of tumor differentiation (well-, moderately- and undifferentiated) (20), which is essential to evaluate the tumor’s growth rate and ability to metastasize (14).

The TIF constitutes the area of the lesion with the greatest depth of invasion and progression into the surrounding tissues (21). In addition, the cells of the TIF have differing molecular characteristics when compared with the cells at the superficial areas of the tumor (10,22). The TIF is considered to be the most representative area of the tumor (23) and is identified by four characteristics; the degree of keratinization, nuclear polymorphism, lymphocytic infiltration and pattern of invasion (PI) (23,24). Of these, the PI is considered to be a good prognostic factor in OSCC (1). To evaluate the severity of the invasion, several morphological criteria exist, associated with certain PIs, according to the following three categories (Fig. 1F–I): i) Islet-infiltrating cells with wide fronts of invasion; ii) thin infiltrating cords; and iii) individual infiltrating cells (1).

In the clinical field, the majority of medical centers base their decisions upon the clinical and pathological information. The TNM stage (T, tumor size; N, regional lymph node compromise; and M, metastasis) (25) and the degree of tumor differentiation (20), combined with the patient’s health status, are the predominant factors that determine the therapeutic strategy. To advance the knowledge of OSCC, numerous pathological and molecular clinical markers have been identified for the prediction of prognosis (1).

## 3. Tumor biomarkers

Transformed neoplastic cells determine the biological behavior of the tumor. Aberrant cells, which posess common features, present a wide range of morphological and functional disorders.

Genetic and epigenetic alterations in OSCC lead to changes that include reduced expression or overexpression of proteins. The accumulation of these changes in oncogenes and tumor suppressor genes may lead to the formation of OSCC. The genes that are critically altered in OSCC include cyclin D1, p53, retinoblastoma, epidermal growth factor receptor, signal transducer and activator of transcription 3, and vascular endothelial growth factor receiver, as well as other molecules (26,27).

### Ki-67 and p53

Ki-67 and p53 are the most commonly used tumor markers for studying cell proliferation. The p53 protein is one of the transcription factors that is implicated in cell cycle control, apoptosis and preservation of genetic stability (28). In addition, the p53 gene is one of the most commonly mutated genes in OSCC with mutations detected in >50% of OSCC cases (29). The activation of p53 has been reported in a number of processes, such as DNA damage, hypoxia and oncogene activation. In addition, p53 protects against tumor formation by preventing the accumulation of cells with DNA damage, which subsequently induces a loss of function in the majority of malignant neoplasms (30). Although not completely understood, Ki-67 is considered to be an important protein in cell division, as it has been observed that the antigen is expressed primarily during the cell cycle stages of G1, S, G2 and M, with a marked emphasis on the M phase. However, Ki-67 expression is not observed during the G0 phase and has a low expression in the G1 and S phases (31). Furthermore, Ki-67 is considered to be one of the best predictors of survival (Fig. 1J and K) (16) and recurrence (5).

### Homeobox (HOX) genes

Recently, novel markers have been used to assess morphogenesis and cell differentiation. Previous studies have demonstrated that the aberrant expression of genes is associated with cancer embryogenesis, particularly the HOX genes that may induce embryological development, as well as contribute to the onset and progression of tumors (32,33). Furthermore, HOX gene overexpression has been associated with carcinogenesis, including head and neck neoplasms (34) and HOXB7, a member of the family of homeodomain transcription factors, is a critical regulator of development, controlling the proliferation and survival of progenitor cells. In OSCC, HOXB7 is overexpressed (Fig. 1L and M) (32), which has been confirmed to be associated with a poor prognosis in OSCC and other types of cancer (32,35).

### Collagen type IV (ColIV)

Infiltration is a key prerequisite for cancer metastasis, making it a significant factor in the prognosis of patients with OSCC (36). For the activation of the process, degradation of the BM must occur between the epithelium and lamina propria, which is located around the nest of cancer cells and blood vessels. The BM has been identified as a crucial structure in the regulation of tumor invasion. Its molecular assembly is a barrier for the invasion of the connective tissue, in particular of the epithelial cells, unless a molecular rupture occurs (37).

ColIV is the most important protein component of the BM and its integrity is altered by the degradation of the BM via matrix metalloproteinases (MMP) 2 and 9 that are present in OSCC (Fig. 1N) (38) and the surrounding tissues (36). Furthermore, MMP 2 and 9 facilitate the development of lymph node metastases (38,39). Therefore, monitoring the changes in the expression of ColIV may have prognostic value in OSCC patients (36,40).

## 4. Tumor microenvironment (TME)

For a number of years, cancer has been considered a cell-autonomous process in which consecutive mutations in the oncogenes and tumor suppressor genes lead to the infinite proliferation of neoplastic cells (41). Thus, cancer therapeutic strategies have been focused and limited on such mutations within the tumor cells (4). However, increasing evidence indicates that the genesis and progression of the tumor is determined by tumor cells as well as by a low TME (42).

Recent findings have indicated that for the effective control of cancer, the genesis and progression of the tumor must not only be considered to be cell-autonomous, but predominantly as a disease that involves complex heterotypic multicellular interactions within the newly formed tissue and the original cancerous tissue. Furthermore, the disease must be considered to be a a systemic, solid-tumor tissue disease rather than a single disease entity. Therefore, the concept of the TME has been proposed as an integral aspect and essential area of cancerous tissues. Recent evidence from a study concerning the TME has emerged, forcing the scientific community to review the basics of cancer biology (43).

**Figure 2:**
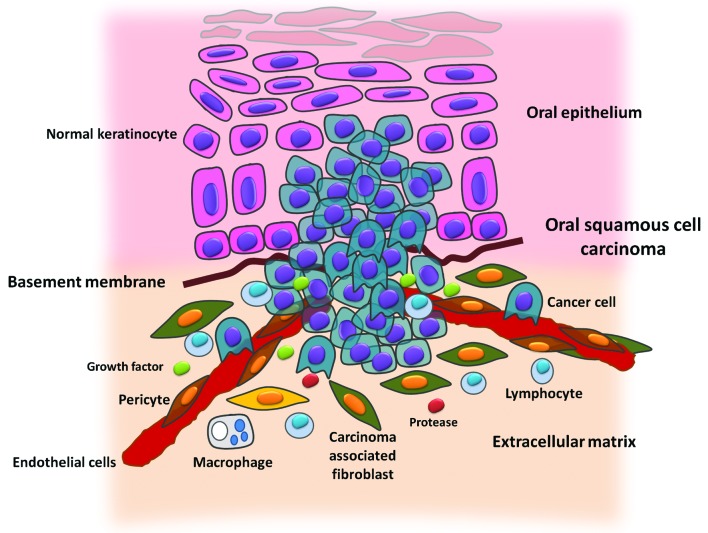
In the tumoral microenvironment (TME), different stromal cells, as well as tumor cells were observed, including vascular and lymphatic endothelial cells, and pericyte support fibroblast innate and adaptive immune cells. Furthermore, the TME contained no cellular components, including the extracellular matrix, growth factors, proteases, protease inhibitors or other signaling molecules that are significant in the reactions of the stroma in the TME (4).

The TME contains numerous types of cells, including fibroblasts, cancer-associated fibroblasts (CAFs), myofibroblasts, smooth muscle cells, endothelial cells and their precursors, pericytes, neutrophils, eosinophils, basophils, mast cells, T and B cells, natural killer cells, and antigen presenting cells, such as macrophages and dendritic cells (Fig. 2).

### CAFs

Despite a marked recruitment of immune cells in the TME, immune cells do not represent the main population of tumor stromal cells; CAFs are the most abundant cells of the TME. CAFs are generally identified by the expression of α-smooth muscle actin, which is similar to the expression of myofibroblasts that occurrs at the site of wound healing and chronic inflammation, however, is absent in normal skin fibroblasts (44,45).

CAFs may be locally differentiated from normal fibroblasts or surrounding stromal stem cells that are derived from the mesenchymal cells of bone marrow, which is recruited by the tumor (46). The tumor stroma is rich in CAFs, which may be scattered or found in the tumor periphery. Certain evidence indicates that CAFs mechanically reshape the extracellular matrix, via the use of proteases, to facilitate the invasion of cancer cells (4). Previous studies have also demonstrated the existence of a molecular dialogue between CAFs and tumor cells, the latter of which secrete interleukin 1α, which stimulates the secretion of chemokine (CC motif) ligand 7 from the CAFs, resulting in tumor progression (6). The increased presence of CAFs observed in OSCC has been associated with a diffuse invasion pattern, preparing the environment for tumor invasion and metastasis (47), and is associated with a poor prognosis (48).

## 5. Conclusion

In conclusion, an association between cell proliferation markers in the basal lamina and connective tissue has been identified in OSCC. In addition, hyperproliferative neoplastic cells may induce ColIV degradation and facilitate tumor invasion. Once installed in the connective tissue, the invading tumor cells may stimulate fibroblasts, which results in an increase in the presence of CAFs. This scenario may be associated with clinical and histopathological characteristics, in terms of a more aggressive stage of disease and a poor differentiation grade of tumor invasion, as well as the decreased survival time of patients with increased rates of cell proliferation, loss of BM integrity and CAF expression within the connective tissue.

Therefore, the comparison of these factors with the survival time of OSCC patients, from the time of histopathological diagnosis, is of interest. The results of the present review may be useful to clarify the tumor-stromal interaction, and its significance regarding the clinical and histological characteristics of OSCC, in order to expand the quantity of specific prognostic factors available as alternatives to the classic TNM.
